# Effects of scapular treatment on chronic neck pain: a systematic review and meta-analysis of randomized controlled trials

**DOI:** 10.1186/s12891-024-07220-8

**Published:** 2024-04-01

**Authors:** Yin Chen, Chunlan Yang, Kailu Nie, Jiapeng Huang, Yun Qu, Tingting Wang

**Affiliations:** 1 Department of Rehabilitation Medicine, West China Hospital of Sichuan University, Chengdu, 610041 Sichuan China #37, Guo Xue Lane,; 2 College of Rehabilitation Medicine, West China Hospital of Sichuan University, Chengdu, 610041 Sichuan China; 3 Sichuan Provincial Key Laboratory of Rehabilitation Medicine, Sichuan University, Chengdu, 610041 Sichuan China

**Keywords:** Chronic neck pain, Scapular treatment, Scapular stabilization exercise, Scapular correctional exercises, Scapulothoracic mobilization

## Abstract

**Background:**

Chronic neck pain (CNP) is a common public health problem that affects daily living activities and quality of life. There is biomechanical interdependence between the neck and scapula. Studies have shown that shoulder blade function might be related to chronic neck pain. We therefore evaluated the effects of scapular targeted therapy on neck pain and function in patients with CNP.

**Methods:**

Databases, including MEDLINE (via PubMed), EMBASE (via Ovid), Ovid, Web of Science, and Scopus, were systematically searched for randomized controlled trials published in English investigating treatment of the scapula for CNP before July 16, 2023.

**Results:**

A total of 313 participants were included from 8 RCTs. Compared with those in the control group, the intervention in the scapular treatment group exhibited greater improvement in pain intensity (standardized mean difference (SMD) = 2.55; 95% CI = 0.97 to 4.13; *P* = 0.002), with moderate evidence. Subgroup analysis for pain intensity revealed a significant difference between the sexes, with only the female population (SMD = 6.23, 95% CI = 4.80 to 7.65) showing better outcomes than those with both sexes (SMD = 1.07, 95% CI = 0.57 to 1.56) (*p* < 0.00001). However, moderate evidence demonstrated no improvement in neck disability after scapular treatment (SMD of 0.24[-0.14, 0.62] of Neck Disability Index or Northwick Park Neck Pain Questionnaire). No effect of scapular treatment was shown on the pressure pain threshold (PPT). The cervical range of motion (CROM) and electromyographic activity of neck muscles could not be conclusively evaluated due to limited support in the articles, and further study was needed. However, the patient’s head forward posture appeared to be corrected after scapular treatment.

**Conclusion:**

Scapular therapy was beneficial for relieving pain intensity in patients with CNP, especially in women. Head forward posture might also be corrected with scapular therapy. However, scapular therapy may have no effect on the PPT or neck disability. However, whether scapular therapy could improve CROM and cervical muscle activation in patients with CNPs had not been determined and needed further study.

**Supplementary Information:**

The online version contains supplementary material available at 10.1186/s12891-024-07220-8.

## Introduction

Neck pain ranks as the second most common skeletal-muscular disease [1], with an age-standardized prevalence rate of 3551.1/100,000 people [2]. Neck pain that lasts for more than three months can be defined as chronic neck pain (CNP) [3]. Compared with people without neck pain, people with neck pain have a reduced ability to perform activities of daily living, poorer quality of life and health, and increased disability as the duration and intensity of pain increase [4–6].

The current view is that neck pain is not only a problem of the cervical spine itself but also a broader area, such as the shoulder blade [7]. An abnormal scapular position and kinematics can lead to functional dynamic chain breaks, increasing the risk of injury to the connected region [8, 9]. From a structural point of view, scapular dysfunction may be associated with neck pain and dysfunction because multiple muscles, such as the levator scapularis and superior trapezius, are attached to both the scapula and the cervical spine. An imbalance of coordination or increased tension in the muscles of the scapular region may directly increase small joint loading in the neck through the common muscles [10]. Compared with healthy people, patients with neck pain tend to exhibit muscle stiffness, fatigue and atrophy in the scapular region [11, 12], leading to abnormal scapular positioning and motor dysfunction [13]. Therefore, subsequent abnormal scapular position or function can in turn increase tissue mechanical sensitivity and neck pressure pain sensitivity [14–16]. Compared to healthy people, people with abnormal scapular positioning exhibit prolonged and repetitive nerve compression and greater neck neural tissue mechanosensitivity, resulting in neck pain [14]. The interaction between neck pain and the abnormal function of the scapula may be the mechanism for the occurrence, maintenance and even aggravation of chronic neck pain [17, 18]. Consequently, focusing on the scapular region in patients with CNP is highly necessary.

Although scapular therapies are recommended for chronic neck pain treatment [19], the effect of scapular therapy on neck pain is still controversial. Several studies have shown that scapular therapy can effectively ameliorate neck pain and neck dysfunction [20–22]. Another study provided "fair" to "good" evidence that treatment targeting scapular kinematics and stability could reduce neck pain severity [23]. However, some studies have shown that this effect is not significant for improving pain intensity [24] or neck function [25]. To our knowledge, a systematic review [26] summarizing the effect of scapular stabilization exercise on CNP has been published, but no effective conclusions have been drawn due to the insufficient quantity and low quality of the literature. A comprehensive meta-analysis to rigorously evaluate the effect of scapular treatment on CNP incidence is lacking; therefore, we aimed to conduct a meta-analysis of randomized controlled trials (RCTs) through multiple literature searches to investigate the potential effects of scapular therapy in reducing pain and neck function in patients with CNP.

## Method

### Protocol and registration

This systematic review and meta-analysis were conducted according to the Preferred Reporting Items for Systematic Reviews and Meta-analyses (PRISMA) reporting guidelines [27]. The study was registered in the International Prospective Register of Systematic Reviews (PROSPERO) (ID: CRD42023303203).

### Selection criteria

The selection criteria followed the PICOS principle. Populations: Patients older than 18 years with chronic neck pain (duration ≥ 3 months) were included. Patients with abnormal posture, position and dyskinesia in the cervical spine and scapula were allowed. Patients with neck pain caused by any of the following disorders were excluded: cervical infection, fracture or tumor, spinal trauma, whiplash injury, radicular pain, disc disease, or neurologic disorders such as stroke. Interventions: Scapular treatment, such as postural correctional exercises (SCE) and scapular stabilization exercise (SSE) or scapulothoracic mobilization (STM), should be implemented. Outcomes: Any study that assessed neck pain and function using relevant scales or instruments could be included. Comparison: The blank control was used as the blank control group. When the experimental group received only scapular therapy, the control group did not receive any intervention. When the control group received other treatments, scapula treatment was added only to the experimental group. Study design: Included studies were clinical randomized controlled trials.

### Study selection

Two authors (KN and CY) separately searched the following electronic databases for articles published before July 16, 2023: MEDLINE (via PubMed), EMBASE, Ovid, Web of Science, and Scopus. The search strategy is provided in the Supplementary material. All the papers were imported into EndNote20 for further screening. After duplicates were removed, two researchers separately assessed the eligibility of the studies. The initial screening was based on the title, abstract or reference type of the articles. Articles not written in English, nonrandomized studies, animal studies, reviews, letters and literature unrelated to the intended topic were excluded. Then, a full-text screening was performed to identify the final inclusion criteria. Disagreements were resolved by a third researcher.

### Outcome measures

This review analysed neck pain intensity utilizing the visual analog scale (VAS) and numerical rating scale (NRS) and cervical disability evaluated by the Neck Disability Index (NDI) or Northwick Park Neck Pain Questionnaire (NPQ) as primary outcomes. The pressure pain threshold (PPT) of the neck muscles, cervical range of motion (CROM), electromyographic activity of the neck muscles, and head forward posture measured by the craniovertebral angle (CVA) were analysed as secondary outcomes.

### Data extraction

Two reviewers (TW and JH) independently extracted the following data: authors and years of the literature; population characteristics; interventions, including type of therapy; duration, frequency and timing of follow-up assessment; and outcomes. The mean changes from baseline to the end of intervention or follow-up were expressed using the means and SDs (the average and standard deviation) directly extracted from the articles or calculated by measurement results before and after treatment using the following formula with *r* = 0.5, according to the *Cochrane Handbook for Systematic Reviews of Interventions* [28]. $SD=\sqrt{{{SD}_{final}}^{2}+{{SD}_{baseline}}^{2}-{2\times r\times SD}_{final}\times {SD}_{baseline}}$ When medians were reported, they were transferred into means and standard deviations (SDs) [29, 30]. When the data were fuzzy or presented as pictures, we emailed the author to ask for data or used a web-based digitizer (WebPlotDigitizer 4.6) to extrapolate the data from the data pictures.

### Risk of bias and methodological quality assessment

The two reviewers mentioned above also assessed the risk of bias for each of the included RCTs using the Cochrane risk-of-bias 2.0 tool [28], which judges articles according to random sequence generation, allocation concealment, blinding of participants and outcome assessment, incomplete outcome data, and selective reporting and classifies each domain as low risk, high risk, or unclear risk. A graphical representation of the risk of bias assessment was generated by RevMan (version 5.4). The Grading of Recommendations, Assessment, Development and Evaluations (GRADE) assessment [31] was applied to rate the quality of evidence considering five aspects: the risk of bias, inconsistency, indirectness, imprecision, publication bias, and other factors, classifying the evidence into high, medium, low, and very low.

### Statistical analysis

Statistical analyses were performed using RevMan 5.4. Source heterogeneity was analysed using the I^2^ statistical test. When *P* < 0.05 or I^2^ > 50%, indicating significant heterogeneity between studies, a random effects model was used. Otherwise, a fixed-effect model was used. When heterogeneity was detected, sensitivity and subgroup analyses were performed by removing one study iteratively to evaluate the robustness of the results.

## Results

### Study selection

**Fig. 1:**
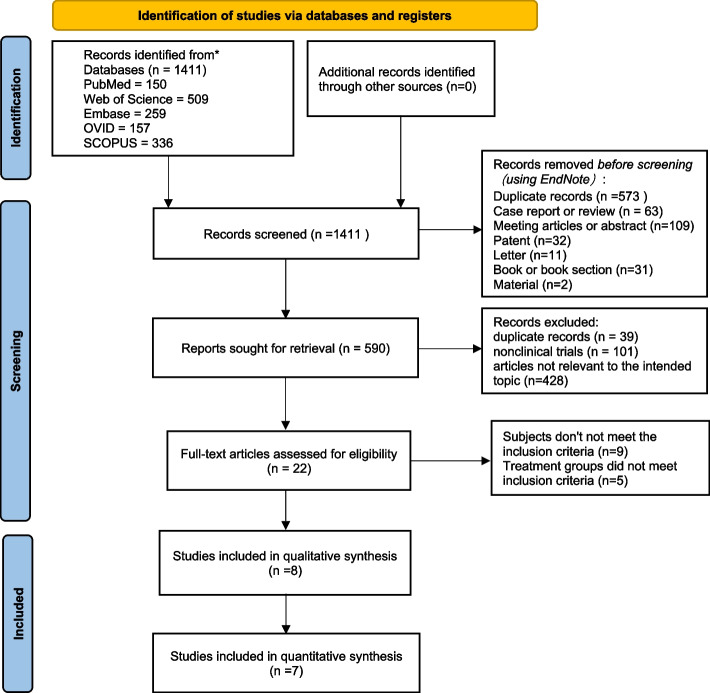
Flow diagram of the study selection process

The PRISMA flowchart shows our process for the inclusion of literature (Fig. 1). A total of 1411 records were retrieved from the database. Duplicate options, nonclinical research articles such as reviews and conference abstracts and irrelevant studies were removed first. Finally, 8 studies [21, 24, 25, 32–36] were selected for further evaluation after eliminating articles that did not meet the inclusion criteria of our study. Due to the lack of detailed data in Yildiz's study [36], only 7 studies were included in the quantitative synthesis.

### Study and patient characteristics

All 8 RCTs [21, 24, 25, 32–36] published in English between 2016 and 2023 were included in the meta-analysis. These studies were conducted in Asia (the Kingdom of Saudi Arabia [24], Korea [32] and Iran [21, 25, 33, 35]) and Europe (Turkey [34, 36]). A total of 313 participants were enrolled, and the sample sizes of these studies ranged from 15 to 87. All the studies [21, 24, 25, 32–36] included patients with CNP that persisted for 3–6 months in the neck region. Some of the neck pain patients in these included studies also had problems with abnormal shoulder and neck posture [21, 25, 32] or scapular movement [24, 34–36]. The included patients were younger than 50 years old, considering the need to minimize the impact of degenerative changes on neck function. All the articles included both men and women, except for two studies [25, 35] that included only females. The detailed characteristics of the eligible studies are shown in Table 1 (at the end of this text).

**Table 1:** Characteristics of the included studies

Author, year	Participants (n, M/F; Age^a^; Onset)	Interventions	Dosage	Outcomes
Alshami, A.M. 2021 [24]	IG (*n* = 20; M/F: 16/4) Age: 33 ± 6 years Onset: 13 ± 10 months CG (*n* = 20; M/F: 15/5) Age:37 ± 7 years Onset: 25 ± 29 months	IG: STM + postural correctional exercises + tape CG: postural correctional exercises + tape	six sessions over 2 to 3 weeks	Pain Intensity (NRS + PPT); NDI; cervical ROM; Scapular ROM
Im, B., et al. 2016 [32]	IG (*n* = 8; M/F: 5/3) Age: 35.5 ± 8.8 years CG (*n* = 7; M/F: 6/1) Age: 35.7 ± 9.8 months	IG: SSE; CG: no intervention	treatments 3 times/week for 4 weeks	Pain Intensity (VAS); NDI; Electro myographic activity of UT, LT and SA; CVA; WHOQOL-BREF
Javdaneh, N. 2021 [33]	IG (*n* = 24; M/F: 14/10) Age: 34.25 ± 8.01 years Onset: 4.25 ± 1.85 years CG (*n* = 24; M/F: 13/11) Age: 32.58 ± 6.37 years Onset: 3.18 ± 1.54 years	IG: neck exercise + SSE; CG: neck exercise	40 to 60 min of training three times a week for 6 weeks	Pain Intensity (VAS); cervical ROM, CVA; SDRI
Özdemir, F., et al., 2021 [34]	IG (*n* = 13; M/F: 6/7) Age: 39.6 ± 13.9 years Onset: 18.4 ± 9.6 months CG (*n* = 11; M/F: 6/5) Age:42.8 ± 13.8 years Onset: 17.1 ± 26.6 months	IG: normal physical therapy + SSE; CG: normal physical therapy	treatments 5 times/week for 3 weeks	Pain Intensity (VAS); NPQ
Shiravi, S. 2019 [25]	IG (*n* = 44; M/F: 0/44) Age: 27.6 ± 2.06 years CG (*n* = 43; M/F: 0/43) Age: 25.11 ± 1.99 years	IG: SSE; CG: no intervention	Three 30-min sessions per week for 6 weeks	Pain Intensity (VAS); muscle strength and Electro myographic activity of UT, MT, LT and SA; Shoulder Proprioception
Shirzadi, Z. 2018 [35]	IG (*n* = 23; M/F: 0/23) Age: 39.82 ± 10.12 years CG (*n* = 23; M/F: 0/23) Age: 39.13 ± 10.50 years	IG: normal physical therapy + STM; CG: normal physical therapy	treatments 5 times/week for 2 weeks	Pain Intensity (NRS); NDI; grip strength; Disabilities of Arm, Shoulder and Hand Questionnaire; NDI
Srikrajang, S. 2023 [21]	IG (*n* = 14; M/F: 6/8) Age: 31.0 (20.8, 38.3) years Onset: 12.0 (6.0, 24.0) months CG (*n* = 14; M/F: 4/10) Age: 31.0 (25.8, 34.3) years Onset: 12.0 (6.0, 24.0) months	IG: SCE CG: no intervention	single treatment	Pain Intensity (NRS + PPT); cervical ROM
Yildiz, T.I. 2018 [36]	IG (*n* = 12) Age: 27.8 ± 8 years CG (*n* = 13) Age:32.8 ± 7.4 years	IG: neck exercise + SSE; CG: neck exercise	treatments every day for 6 weeks	Pain Intensity (VAS); NDI; Scapular ROM

*M* Male, *F* Female, *IG* Intervention group, *CG* Control group

*SSE* Scapular stabilization exercise, *SCE* Scapular correctional exercise, *STM* Scapulothoracic mobilization

*PPT* Pressure pain threshold, *VAS* Visual analogue scale, *NRS* Numeric rating scale

*NDI* Neck disability index, *NPQ* Northwick Park Neck Pain Questionnaire

*CVA* Craniovertebral angle, *ROM* Range of motion

*UT* Upper trapezius, *MT* Middle trapezius, *LT* Low trapezius, *SCM* Sternocleidomastoid muscle, *SA* Serratus anterior

*WHOQOL-BREF* World Health Organization Quality of Life Assessment-BREF

### Interventions

The scapular treatment in the included studies included active exercises, such as scapular correctional exercises [21] and stability exercises[25, 32–34, 36], as well as passive exercises, such as scapulothoracic mobilization [24, 35]. In the control group of the 6 articles [24, 33–36], certain treatment measures were adopted, such as cervical stability exercises, postural correction training, elastic band therapy or normal physical therapy, while treatment of the scapula was added to the experimental group on the basis of the control group. The other 3 studies [21, 25, 32] set blank controls as control groups for scapular treatment.

### Risk assessment

**Fig. 2:**
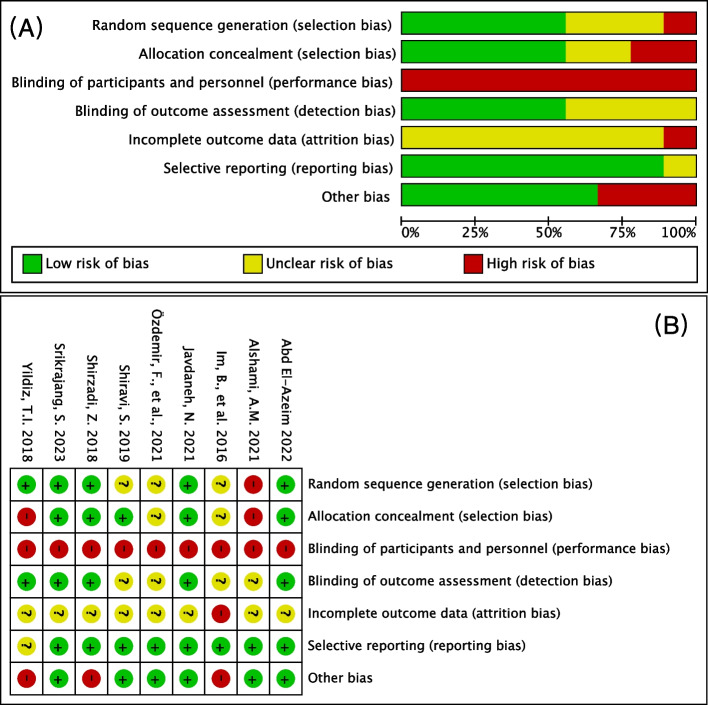
(**A**) Risk of bias graph and (**B**) risk of bias summary

The risk assessment is shown in Fig. 2. As 3 studies [25, 32, 34] did not describe the process of randomization and 2 studies [32, 34] did not describe the allocation process, they were considered to have unclear risk. Alshami AM’s study [24] used alternate randomization, which was the wrong method, leading to a high risk of randomization and allocation. All the studies [21, 24, 25, 32–36] were high risk for participant blinding, as none of the included studies met the criteria for participant blinding. Whether evaluators knew the allocations of patients was not clear in 4 studies [24, 25, 32, 34], which were believed to have an unclear risk. None of the included studies had follow-up data, so all of them were of unclear risk [21, 24, 25, 32–36]. Three studies [32, 35, 36] were considered high risk because of unclear or insufficient baseline information and outcomes.

### Effects of interventions

#### Pain intensity (VAS or NRS)

**Fig. 3:**
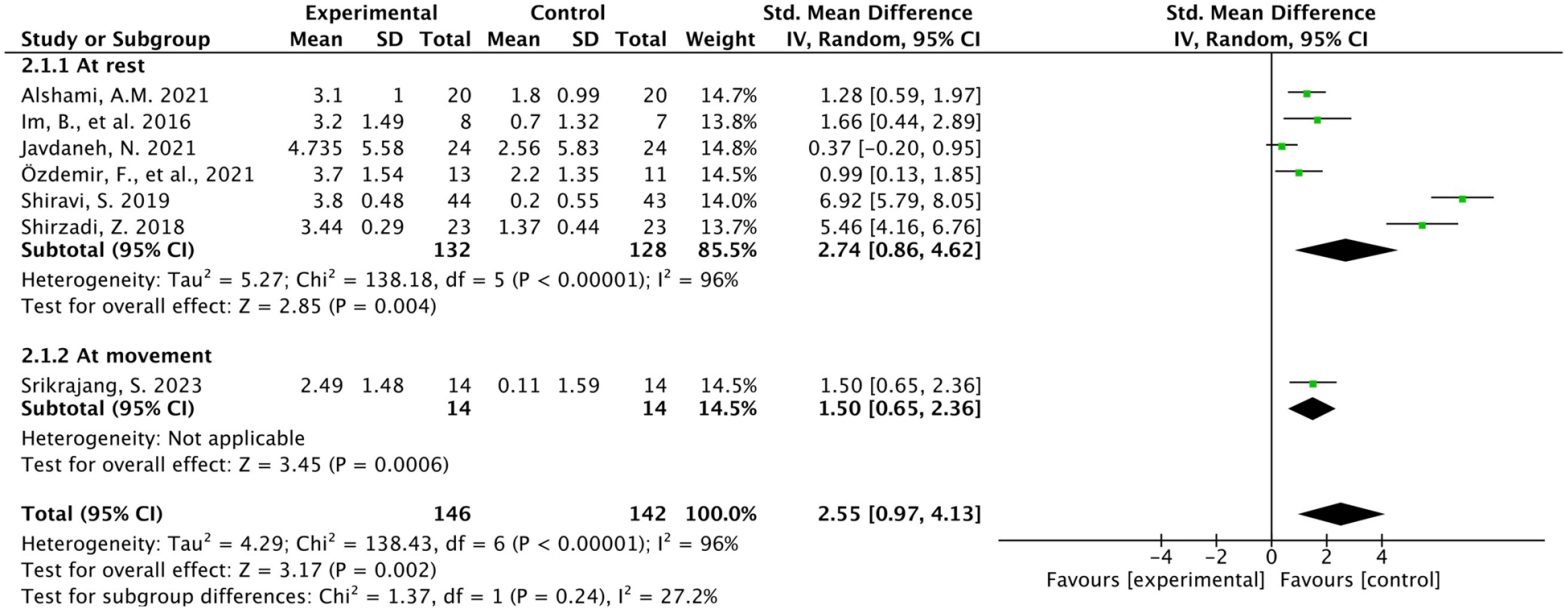
Forest plots of pain intensity (VAS or NRS) in the intervention and control groups

Eight studies [21, 24, 25, 32–36] measured pain intensity before and after inventions using the VAS or NRS, but one study [36] did not report available data. Six studies [24, 25, 32–35] were included in the meta-analysis; six studies were assessed at rest, and one study [21] was at the end range of maximum active cervical rotation. The results showed that the standardized mean difference (SMD) was 2.55 (95% CI, 0.97 to 4.13; *P* = 0.002), with high heterogeneity (Chi^2^ = 138.43, *P* < 0.00001; I^2^ = 96%) (Fig. 3) in patients with neck pain in the intervention group vs. the control group. The aggregated results did not change after excluding studies with high heterogeneity iteratively (Table 2).

**Table 2:** Results of sensitivity analyses after removing one study iteratively

Excluded trials	Test for effect		Heterogeneity		
	SMD (95%CI)	*p* value	Chi^2^	*p* value	I^2^
Alshami AM. 2021 [24]	2.78 [0.82, 4.75]	0.005	136.53	< 0.00001	96%
Im B 2016 [32]	2.70 [0.91, 4.49]	0.003	138.42	< 0.00001	96%
Javdaneh N 2021 [33]	2.94 [1.12, 4.76]	0.002	108.15	< 0.00001	95%
Özdemir F 2021 [34]	2.82 [0.96, 4.69]	0.003	135.36	< 0.00001	96%
Shiravi S 2019 [25]	1.79 [0.69, 2.89]	0.001	50.29	< 0.00001	90%
Shirzadi Z 2018 [35]	2.09 [0.54, 3.63]	0.008	104.53	< 0.00001	95%
Srikrajang S 2023 [21]	2.74 [0.86, 4.62]	0.008	138.18	< 0.00001	96%

*SMD* Standardized mean difference

To analyse the heterogeneity, a subgroup meta-analysis was conducted on disease characteristics, sex, and scapular interventions. The subgroup analysis of CNP patients with abnormal posture and STM showed no significant differences (SMD = 3.36 (-0.07, 6.78); SMD = 3.33 (-0.76, 7.43); Table 3). According to the guidelines for interpreting subgroup analysis [37], we concluded that the treatment effect was very similar among the subgroups given that the confidence intervals of the subgroup analyses completely overlapped each other. Therefore, no differences in disease characteristics or interventions were found within groups (*p* = 0.69, 0.31). Female-only studies performed better effectiveness than did studies that included men and women (*p* < 0.00001; Table 3).

**Table 3:** Subgroup analysis of pain intensity (VAS or NRS)

Subgroups	Variables	Studies, n	Patients, n	SMD (95%CI)	Test for subgroup difference	
					Chi^2^	*p* value
Disease characteristics	CNP with abnormal posture	3	130	3.36 [-0.07, 6.78]	0.16	0.69
	CNP with scapular dyskinesia	3	110	2.51 [0.28, 4.74]		
	Total	6	240	2.94 [1.12, 4.76]		
gender	Female only	2	133	6.23 [4.80, 7.65]	44.82	< 0.00001
	Both male and female	5	155	1.07 [0.57, 1.56]		
	Total	7	288	2.55 [0.97, 4.53]		
inventions	SSE	4	174	3.32 [0.77, 5.88]	2.35	0.31
	SCE	1	28	1.50 [0.65, 2.36]		
	STM	2	86	3.33 [-0.76, 7.43]		
	Total	7	288	3.05 [1.46, 4.64]		

*CNP* Chronic neck pain, *SSE* Scapular stabilization exercise, *SCE* Scapular correctional exercises, *STM* Scapulothoracic mobilization, *SMD* Standardized mean difference

Yildiz’s research [36], which did not provide specific data, suggested that adding extra scapular treatment could significantly improve pain intensity but was not different from what was observed in the group receiving neck-focused exercise and scapular stabilization training.

#### Neck disability status (NDI or NPQ)

**Fig. 4:**
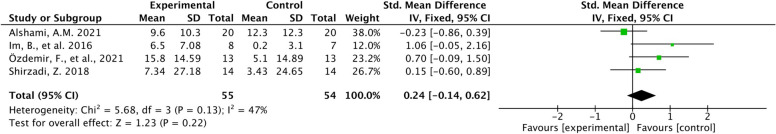
Forest plots of neck function (NDI or NPQ) in patients with neck pain in the intervention and control groups

Five studies [24, 32, 34–36] evaluated the outcome of neck function with multiple scales, 3 studies used the NDI [24, 32, 35], and 1 study used the NPQ[34]. The results of the quantitative synthesis did not reveal a significant effect on neck function, with an SMD of 0.24 (95% CI, -0.14 to 0.62; *P* = 0.22) and significant heterogeneity (Chi^2^ = 5.68, *P* = 0.13; I^2^ = 47%). Figure 4).

#### PPT

Two studies [21, 24] reported no significant effect of scapular treatment on the PPT. We did not merge the articles because of the limitations of the number of articles and the different positions of the ppt measurements (one measured in the UT [21] and the other used the average PPT of the cervical spine, trapezius and levator scapulae muscles [24]).

#### CROM

CROM was presented in 3 studies [21, 24, 33], all of which had different results. According to the findings of Javdaneh N's study [33], the scapular treatment group exhibited greater improvements in the CROM at cervical flexion (*p* = 0.024) and extension (*p* = 0.025) than did the control group. Shizadi Z et al. [21] found that scapular treatment significantly improved the CROM of neck rotation (*p* = 0.006, 0.016), while the control group did not significantly differ before or after treatment. However, Alshami AM [24] reported no difference (*p* > 0.05) in the CROM of any cervical spine movements, including flexion, extension, left rotation, right rotation, or left and right flexion, between the intervention and control groups.

#### Electromyographic activity of neck muscles

Im B et al. [32] suggested that the activation of the upper trapezius muscle decreased (*P* < 0.05) and that the activity of the serratus anterior muscle increased (*P* < 0.05). There was no significant improvement in the control group. Shravi [25] reported that neither the test group nor the control group showed improvement in the electromyographic activity of the trapezius and serratus anterior muscles (*P* > 0.05).

#### Head forward posture

Both Im B et al. [32] and Javdaneh’s study [24] showed that CVA was greatly improved in the experimental group, while the control group did not show improvement.

#### GRADE analysis of the evidence

Moderate and low quality evidence was found for pain intensity, neck function, CROM, electromyographic activity of neck muscles and CVA (a detailed GRADE evaluation of evidence grades of pain intensity and neck disability is provided in the Supplementary material).

## Discussion

This was the first systematic review and meta-analysis of RCTs analysing the effect of scapular treatment on improving CNP incidence. There was moderate-quality evidence that scapular treatment alone could reduce subjective pain intensity in patients with CNP. The outcome of our study was persuasive, as the experimental groups in the included articles only received scapular therapy in combination with the control group. Excess activation and decreased control or weakness of scapulothoracic muscles, such as the trapezius and serratus anterior, may lead to neck pain [38]; thus, the mechanism by which scapular therapy improves neck pain may stem from changes in muscle activity in the neck [39]. Scapular treatment, including stability exercises, corrective exercises and scapulothoracic mobilization, could reduce the tension of cervicoscapular muscles [10], strengthen the neck muscle strength [40], and restore the normal biomechanical structure of the neck [41], thus decreasing abnormal loading in the neck region and reducing neck pain. Subgroup analysis indicated that postural abnormalities or scapular dyskinesia did not influence the effectiveness of scapular treatment for pain intensity in patients with CNP, revealing the general applicability of scapular therapy for people with CNP. In addition, SEE, SCE and STM had the same ability to relieve pain in the CNP population, which demonstrated the effectiveness of different scapular treatment methods. Notably, females with CNP seemed to be better treated with scapular treatment, which was a valuable finding, as females have shown a trend toward a greater incidence of CNP than males in recent years [42].

As there was a high degree of heterogeneity in this study, sensitivity analyses were performed, revealing no changes in the results. Subsequent subgroup analysis of patient characteristics and intervention methods did not reveal reduced heterogeneity, whereas reduced heterogeneity was found for the gender subgroups, which revealed that the source of heterogeneity might be the sex composition of the included patients. In addition, we did not analyse specific interventions, such as exercise posture, frequency, or treatment cycle, which might also be responsible for the heterogeneity.

Two studies [21, 24] showed no effect of scapular therapy on PPT improvement in CNP patients, which was inconsistent with the findings of previous studies [16, 20, 38]. The reasons might be associated with differences in PPT measurement tools and locations, as well as with interventions [24]. Therefore, the effect of scapular therapy on improving cervical tissue mechanical sensitivity needs further study.

Our results showed that scapular therapy alone did not seem to improve cervical disability. In addition, no definitive conclusions could be drawn about whether scapular therapy improved cervical muscle activation or CROM because the amount of literature was limited and because the conclusions of these studies were not uniform. Therefore, we believe that scapular therapy alone has little or no efficacy in improving neck function.

Scapular exercise therapy has now been added to head forward posture training as level 1b evidence [43]. Our study also reconfirmed that scapular therapy could change head forward posture through the limited available literature. Although our study did not find that patients with CNP with scapular dysfunction could benefit better from scapular therapy, it is undisputed that scapular training is necessary to improve scapular positional abnormalities and dyskinesia [44]. Therefore, we prefer to recommend the inclusion of scapular therapy for patients with CNP associated with scapular abnormalities.

According to an international survey, physiotherapists most often use exercise therapy and manipulation therapy for neck pain; therefore, we conclude that the use of scapular exercise and mobilization therapy for the treatment of CNP is readily acceptable and achievable for clinical application [45]. The main purpose of this study was to investigate the effectiveness of scapular therapy alone; therefore, articles combining scapular treatment with other therapies were not included or analysed. However, some studies have reported the effects of scapular therapy in combination with upper limb proprioceptive training [40], thoracic extension exercises [46], and cognitive functional therapy [47] for the treatment of CNP. The results showed that, compared to normal neck exercise, scapular treatment combined with other treatments could improve pain and neck function. We believed that the effects of the combination of scapular treatment and other treatments might be the direction and focus of further research.

There were several limitations of the present study. 1) None of the included studies had a follow-up period, so the long-term effect of interventions on the scapula could not be evaluated. 2) There was a lack of evidence for the effects of scapular therapy on cervical muscle strength and quality of life in patients with CNP. 3) All the participants were younger, with an average age ranging from 25.11 to 39.82 years; therefore, the effects of scapular treatment on older populations could not be determined.

## Conclusion

There was moderate-quality evidence that scapular therapy was highly beneficial for improving pain intensity in CNP patients, especially in female patients. The head forward posture also appeared to benefit from scapular treatment. However, the PPT did not improve, and moderate-quality evidence suggested that neck disability assessed using the NDI or NPQ did not improve after scapular treatment. The improvement in CROM and cervical muscles activation in patients with CNP from scapular therapy was uncertain.

### Supplementary Information

**Additional file 1.**

## Data Availability

The datasets supporting the conclusion of this article are included within the article.

## Abbreviations

CNP: Chronic neck pain
RCTs: Randomized controlled trials
SCE: Postural correctional exercises
SSE: Scapular stabilization exercise
STM: Scapulothoracic mobilization
VAS: Visual analog scale
NRS: Numerical rating scale
NDI: Neck Disability Index
NPQ: Northwick Park Neck Pain Questionnaire
PPT: Pressure pain threshold
CROM: Cervical range of motion
CVA: Craniovertebral angle
SD: Standard deviation
GRADE: Grading of Recommendations, Assessment, Development and Evaluations
SMD: Standardized mean difference
