# Supplementary material for: Effects of scapular treatment on chronic neck pain: a systematic review and meta-analysis of randomized controlled trials
Source: BMC Musculoskelet Disord. 2024 Apr 1;25:252. doi: 10.1186/s12891-024-07220-8 (PMC10983729; doi:10.1186/s12891-024-07220-8)
Supplement: Supplementary file 1 — Additional file 1. [file 12891_2024_7220_MOESM1_ESM.docx]

**Search strategy**

| Database | Strategy (before July 16, 2023) |
| --- | --- |
| PubMed  150 | ( "Neck Pain/nursing"[Mesh] OR "Neck Pain/prevention and control"[Mesh] OR "Neck Pain/rehabilitation"[Mesh] OR "Neck Pain/therapy"[Mesh] OR "Neck Pain*"[Title/Abstract] OR "Pain*, Neck"[Title/Abstract] OR "Neck Ache*"[Title/Abstract] OR "Cervicalgia*"[Title/Abstract] OR "Cervicodynia*"[Title/Abstract] OR "Neckache*"[Title/Abstract] OR "Cervical Pain*"[Title/Abstract] OR neck pain[Title/Abstract] OR head forward[Title/Abstract] OR forward head[Title/Abstract] OR upper crossed[Title/Abstract] OR neck function disability[Title/Abstract]) AND (scapulo-thoracic[Title/Abstract] OR scapulothoracic[Title/Abstract] OR scapular-focused[Title/Abstract] OR scapula*[Title/Abstract] OR shoulder balde*[Title/Abstract]) AND (Treatment[Title/Abstract] OR intervention[Title/Abstract] OR massage[Title/Abstract] OR exercise[Title/Abstract] OR physiotherapy[Title/Abstract] OR physical therapy[Title/Abstract] OR manual therapy[Title/Abstract] OR therapy[Title/Abstract] OR stretch*[Title/Abstract] OR stabilisation[Title/Abstract] OR stabilization[Title/Abstract] OR training[Title/Abstract] OR mobilisation[Title/Abstract] OR mobilization[Title/Abstract]) |
| Web of Science  509 | (TI=(("Neck Pain*" OR "Pain*, Neck" OR "Neck Ache*" OR "Cervicalgia*" OR "Cervicodynia*" OR "Neckache*" OR "Cervical Pain*" OR neck pain OR head forward OR forward head OR upper crossed OR neck function disability) AND (scapulo-thoracic OR scapulothoracic OR scapular-focused OR scapula* OR shoulder balde*) AND (Treatment OR intervention OR massage OR exercise OR physiotherapy OR physical therapy OR manual therapy OR therapy OR stretch* OR stabilisation OR stabilization OR training OR mobilisation OR mobilization))) OR AB=(("Neck Pain*" OR "Pain*, Neck" OR "Neck Ache*" OR "Cervicalgia*" OR "Cervicodynia*" OR "Neckache*" OR "Cervical Pain*" OR neck pain OR head forward OR forward head OR upper crossed OR neck function disability) AND (scapulo-thoracic OR scapulothoracic OR scapular-focused OR scapula* OR shoulder balde*) AND (Treatment OR intervention OR massage OR exercise OR physiotherapy OR physical therapy OR manual therapy OR therapy OR stretch* OR stabilisation OR stabilization OR training OR mobilisation OR mobilization)) |
| Embase  259 | 1 neck pain.mp. or exp neck pain/ |
|  | 2 ("Neck Pain*" or "Pain*, Neck" or "Neck Ache*" or "Cervicalgia*" or "Cervicodynia*" or "Neckache*" or "Cervical Pain*" or neck pain or head forward or forward head or upper crossed or neck function disability).ab,kw,ti. |
|  | 3 (scapulo-thoracic or scapulothoracic or scapular-focused or scapula* or shoulder balde*).ab,kw,ti. |
|  | 4 (Treatment or intervention or massage or exercise or physiotherapy or physical therapy or manual therapy or therapy or stretch* or stabilisation or stabilization or training or mobilisation or mobilization).ab,kw,ti. |
|  | 5 1 or 2 |
|  | 6 3 and 4 and 5 |
| Ovid  157 | 1 neck pain.mp. or exp Neck Pain/ |
|  | 2 ("Neck Pain*" or "Pain*, Neck" or "Neck Ache*" or "Cervicalgia*" or "Cervicodynia*" or "Neckache*" or "Cervical Pain*" or neck pain or head forward or forward head or upper crossed or neck function disability).ab,kf,ti. |
|  | 3 (scapulo-thoracic or scapulothoracic or scapular-focused or scapula* or shoulder balde*).ab,kf,ti. |
|  | 4 (Treatment or intervention or massage or exercise or physiotherapy or physical therapy or manual therapy or therapy or stretch* or stabilisation or stabilization or training or mobilisation or mobilization).ab,kf,ti. |
|  | 5 1 or 2 |
|  | 6 3 and 4 and 5 |
| SCOPUS  336 | ( TITLE-ABS-KEY ( "Neck Pain*" OR "Pain*, Neck" OR "Neck Ache*" OR "Cervicalgia*" OR "Cervicodynia*" OR "Neckache*" OR "Cervical Pain*" OR "neck pain" OR "head forward" OR "forward head" OR "upper crossed" OR "neck disability" ) ) AND ( TITLE-ABS-KEY ( scapulo-thoracic OR scapulothoracic OR scapular-focused OR scapula* OR "shoulder balde*" ) ) AND ( TITLE-ABS-KEY ( treatment OR intervention OR massage OR exercise OR physiotherapy OR therapy OR stretch* OR stabilisation OR stabilization OR training OR mobilisation OR mobilization ) ) |

**GRADE evaluation of evidence grades of pain intensity and neck disability.**

| **Certainty assessment** | | | | | | | **№ of patients** | | **Effect** | **Certainty** |  |
| --- | --- | --- | --- | --- | --- | --- | --- | --- | --- | --- | --- |
| **№ of studies** | **Study design** | **Risk of bias** | **Inconsistency** | **Indirectness** | **Imprecision** | **Other considerations** | **IG** | **CG** | **Absolute (95% CI)** |  |  |
| **VAS or NPRS** | | | | | | | | | | | |
| 7 | randomised trials | not serious | serious^a^ | not serious | not serious | none | 146 | 142 | SMD **2.55 higher** (0.97 higher to 4.13 higher) | ⨁⨁⨁◯ Moderate |  |
| **NDI or NPQ** | | | | | | | | | | | |
| 4 | randomised trials | not serious | not serious | not serious | serious^b^ | none | 55 | 54 | SMD **0.24 higher** (0.14 lower to 0.62 higher) | ⨁⨁⨁◯ Moderate |  |
| **PPT** | | | | | | | | | | | |
| 2 | randomised trials | not serious | not serious | not serious | serious^b^ | none | 34 | 34 | - | ⨁⨁⨁◯ Moderate |  |
| **CROM** | | | | | | | | | | | |
| **3** | randomised trials | not serious | serious^c^ | not serious | serious^b^ | none | 58 | 58 | - | ⨁⨁◯◯ Low |  |
| **Electromyographic activity of neck muscles** | | | | | | | | | | | |
| **2** | randomised trials | not serious | serious^c^ | not serious | serious^b^ | none | 52 | 50 | - | ⨁⨁◯◯ Low |  |
| **CVA** | | | | | | | | | | |  |
| 2 | randomised trials | not serious | serious^a^ | not serious | serious^b^ | none | 32 | 31 | - | ⨁⨁◯◯ Low |  |

**IG:** intervention group**; CG:** control group; **CI:** confidence interval; **SMD:** standardised mean difference; **PPT:** pressure pain threshold; **VAS:** visual analogue scale; **NPRS:** numeric pain rating scale; **NDI:** neck disability index; **NPQ:** Northwick Park Neck Pain Questionnaire; **CVA:** craniovertebral angle; **ROM:** range of motion;

Explanations

a. Downgraded one level for serious inconsistency: The high heterogeneity with I² >50%.

b. Downgraded one level for serious inconsistency: Less than 150 participants.

c. Downgraded one level for serious inconsistency: Researches suggest the opposite.
